# The Role of *Helicobacter pylori* Seropositivity in Insulin Sensitivity, Beta Cell Function, and Abnormal Glucose Tolerance

**DOI:** 10.1155/2014/870165

**Published:** 2014-03-25

**Authors:** Lou Rose Malamug, Rudruidee Karnchanasorn, Raynald Samoa, Ken C. Chiu

**Affiliations:** ^1^Department of Clinical Diabetes, Endocrinology and Metabolism, City of Hope National Medical Center, 1500 East Duarte Road, Duarte, CA 91010, USA; ^2^Division of Endocrinology, Metabolism and Nutrition, Department of Internal Medicine, Harbor-UCLA Medical Center, Torrance, CA 90502, USA

## Abstract

Infection, for example, *Helicobacter pylori* (*H. pylori*), has been thought to play a role in the pathogenesis of type 2 diabetes mellitus (T2DM). Our aim was to determine the role of *H. pylori* infection in glucose metabolism in an American cohort. We examined data from 4,136 non-Hispanic white (NHW), non-Hispanic black (NHB), and Mexican Americans (MA) aged 18 and over from the NHANES 1999-2000 cohort. We calculated the odds ratios for states of glucose tolerance based on the *H. pylori* status. We calculated and compared homeostatic model assessment insulin resistance (HOMA-IR) and beta cell function (HOMA-B) in subjects without diabetes based on the *H. pylori* status. The results were adjusted for age, body mass index (BMI), poverty index, education, alcohol consumption, tobacco use, and physical activity. The *H. pylori* status was not a risk factor for abnormal glucose tolerance. After adjustment for age and BMI and also adjustment for all covariates, no difference was found in either HOMA-IR or HOMA-B in all ethnic and gender groups except for a marginally significant difference in HOMA-IR in NHB females. *H. pylori* infection was not a risk factor for abnormal glucose tolerance, nor plays a major role in insulin resistance or beta cell dysfunction.

## 1. Introduction

Diabetes has been identified as a risk factor for* Helicobacter pylori* (*H. pylori*) infection [1]. Compared to subjects without diabetes, the eradication rate of* H. pylori* using the standard regimens was less than satisfactory in patients with type 2 diabetes mellitus (T2DM) [2]. Thus, a potential relationship between* H. pylori* infection and diabetes is highly suspected. Furthermore, diabetes has been acknowledged as an extragastric manifestation of* H. pylori* infection. Improvement of diabetes with eradication of insulin resistance has been reported after successful treatment of* H. pylori* infection [3], although others reported no impact on glycemic control but a significant increase in body mass index after eradication of* H. pylori* infection from a study of 174 patients with T2DM [4]. These reports suggested a strong interaction between* H. pylori* infection and T2DM.

Inflammation has been demonstrated to play a major role in the pathogenesis of T2DM and insulin resistance. Although various causes can lead to inflammation, infection is one of the well-established causes of inflammation. Furthermore, infection has been suspected as a contributing cause among the multifactorial etiologies of T2DM.* H. pylori* is a gram-negative, spiral-shaped bacterium that is associated with chronic gastritis, peptic ulcer disease, gastric adenocarcinoma, and type B low-grade mucosa-associated lymphoma.* H. pylori* infection is associated with increased oxidative stress [5]. The importance of the role of oxidative stress in the development of insulin resistance has been well recognized [6]. Furthermore, oxidative stress has been increasingly implicated in the deterioration of pancreatic islet function [7]. Eradication of infection in* H. pylori*-infected patients improves oxidative stress [8]. Thus,* H. pylori* infection could play a role in the pathogenesis of T2DM.

To further elucidate the relationship of* H. pylori* and glucose metabolism, this study examined the data derived from the National Health and Nutrition Examination Survey (NHANES) 1999-2000. To our knowledge, this is the very first study that examined the role of* H. pylori* infection in abnormal glucose tolerance, insulin resistance, and beta cell function in multiple racial/ethnic groups in a much larger sample size than previously reported.

## 2. Methods

NHANES is a program of the National Center for Health Statistics (NCHS) that was designed to assess the health and nutritional status of adults and children in the United States. Each year a nationally representative sample of approximately 5000 civilian noninstitutionalized individuals in the United States is surveyed. A complex, stratified, multistage probability cluster sampling design was used with oversampling of non-Hispanic blacks and Hispanics. The survey consists of interviews and physical examinations. The examinations include laboratory tests. We have complied with the recommendations of the Declaration of Helsinki. The study was approved by the Research Ethics Review Board of the National Center for Health Statistics, Center of Disease Control.

### 2.1. Study Cohort

This study included subjects aged 18 and over with reported* H. pylori* titer, diabetes status by self-report, HbA1c, and plasma glucose level. We examined data from 1,949 non-Hispanic white (NHW), 853 non-Hispanic black (NHB), and 1,334 Mexican Americans (MA) from the NHANES 1999-2000 cohort. A subset of subjects with fasting glucose and insulin concentrations were included in the analysis of insulin sensitivity and beta cell function.* H. pylori* status was only assessed in the NHANES 1999-2000 cohort, but not in before or after this period.

### 2.2. *H. pylori* Status

*H. pylori* status was defined by the titer of* H. pylori* antibody using the Wampole Laboratories* H. pylori* IgG Enzyme-Linked Immunosorbent Assays (ELISA) for the detection and qualitative determination of IgG antibodies to* H. pylori* in human serum. A value <0.90 is considered negative for the presence of detectable IgG antibody and values greater than 1.10 indicate the presence of detectable IgG antibody. Those with values between 0.91–1.09 were considered equivocal and excluded from analysis.

### 2.3. Status of Glucose Tolerance

Subjects with known type 1 diabetes were excluded from the study. Diabetes was defined as taking oral diabetic medication(s) or insulin, fasting plasma glucose ≥7.0 mmol/L (126 mg/dL), or HbA1c ≥48 mmol/mol (6.5%). Abnormal glucose tolerance included diabetes as described above, fasting plasma glucose levels between 5.5 and 7.0 mmol/l (100 and 126 mg/dL), and HbA1c between 39 and 46 mmol/mol (5.7% and 6.4%). Normal glucose was defined as not taking any oral diabetic medication or insulin with fasting plasma glucose level <5.5 mmol/l (100 mg/dL) and HbA1c <39 mmol/mol (5.7%).

### 2.4. Assessment of Insulin Sensitivity and Beta Cell Function

To avoid skewness in insulin sensitivity and beta cell function, we only included the subjects without diabetes who fasted for at least 8 hours and had fasting plasma glucose and insulin levels available. Based on the homeostatic model assessment (HOMA), we calculated IR (HOMA-IR) and beta cell function (HOMA-B) in subjects without diabetes. HOMA-IR was calculated using the equation (fasting insulin in mU/L × fasting glucose mmol/L)/22.5. HOMA-B was calculated using the equation (fasting insulin in mU/L × 20)/(fasting glucose in mmol/L −3.5).

### 2.5. Statistical Analysis

Differences in continuous variables between the groups of subjects were tested with one-way ANOVA. Differences in proportions were evaluated by a the Chi-square test. Using the Pearson Chi-square test, we calculated the odds ratios for states of glucose tolerance (normal glucose tolerance versus abnormal glucose tolerance, including diabetes) based on the* H. pylori* status by gender and ethnicity. We also compared HOMA-IR and HOMA-B in subjects without diabetes based on the* H. pylori* status. The results were adjusted for age and BMI and also for additional covariates, including poverty index, education, alcohol consumption, tobacco use, and physical activity. The poverty index was computed as the ratio of family income versus the poverty threshold set by the Census Bureau. A continuous combined physical activity score expressed in MET-minutes/wk was obtained for each subject based on average level of physical activity each day. A *P* value less than 0.05 was considered significant. SYSTAT 11.0 for Windows package from SPSS, Inc. (Chicago, IL) was used for statistical analysis.

## 3. Results

The characteristics of the studied subjects were showed in Table 1. Females accounted for a little more than 50% in each racial/ethnic group. Mean age ranged from 43 to 50 years old. The seropositive rate for* H. pylori* was much higher in NHB (54%) and MA (63%) than in NHW (23%, *P* < 0.0001). The prevalence of abnormal glucose tolerance was much higher in NHB (37%) than in NHW and MA (29% and 31%, resp.; *P* < 0.0001).

The subjects with positive* H. pylori* serology were older and had higher HbA1c as compared to those with negative* H. pylori *serology (Table 2).* H. pylori* serology status had no impact on BMI with an exception in MA male subjects. Positive* H. pylori* serology status was associated with abnormal glucose tolerance except in NHB females (*P* = 0.12) and MA males (*P* = 0.05) as shown in Tables 2 and 3 (Model 1). Since the* H. pylori* positive subjects were older than those with negative serology, we first took into account the impact of age and BMI (Model 2) and then age, body mass index, poverty index, education, alcohol consumption, tobacco use, and physical activity (Model 3). No association of* H. pylori *serology status with abnormal glucose tolerance was found in both Models 2 and 3.

To further explore the role of* H. pylori* serology status on glucose metabolism, we compared insulin sensitivity (HOMA-IR) and beta cell function (HOMA-B) in a subset of glucose tolerant subjects who had both fasting plasma glucose and insulin concentration available. No difference in HOMA-IR was found in all ethnic and gender groups based on the* H. pylori* status (Table 4). In contrast to a trend of more insulin resistance that was noted in the seropositive subjects than seronegative subjects in most racial/ethnic and gender groups, NHB female and MA female subjects showed a reverse trend. Due to a drastic difference in age between seronegative and seropositive NHB female subjects (37 ± 16 versus 49 ± 19 years old, resp., *P* < 0.001) and a significant correlation of HOMA-IR with age (*r* = 0.1510, *P* = 0.03), the adjusted difference in HOMA-IR was significant in the NHB female subjects (Table 4). However, no difference was found after adjustment of covariables in other racial/ethnic groups and genders.

Regarding beta cell function, HOMA-B differed only in MA female subjects based on* H. pylori *status (*P* = 0.001, Table 5). However, after adjustment for age and BMI and also adjustment for age and BMI and also for all covariates, no difference was found in HOMA-B in all racial/ethnic and gender groups. Thus,* H. pylori* infection was not a risk factor for beta cell dysfunction.

## 4. Discussion

In this cross-sectional multiethnic group study, we found that the seropositive rate was much higher in the NHB and MA subjects (54% and 63%, resp.) than in NHW subjects (23%). Our data were consistent with the report of 25% in NHW, 58% in NHB, and 70% in MA from the Multiethnic Study of Atherosclerosis [9], although the seropositive rate was much lower than 92% in MA from the Sacramento Area Latino Study on Aging [10]. The latter was mainly obtained from a much older population with a mean age of 69 years old compared to a mean age range of 43–50 years old in the present study. As noted, seropositive rate increases with aging in a mixed American population [11], in a Mexican American population from San Antonio, Texas [12], and also in an Australian population [13]. Thus, the current sample set is a representative American population.

As compared to other studies, we examined the association of* H. pylori *serological status with abnormal glucose tolerance, but not with overt T2DM. Insulin resistance and beta cell dysfunction are the two major defects leading to T2DM. It has been well-recognized that these two defects are present in subjects with abnormal glucose tolerance before the development of overt T2DM in both prospective longitudinal and cross-sectional epidemiological studies. Thus, we chose abnormal glucose tolerance as the phenotype in this study. As compared to abnormal glucose tolerance, there were only 422 subjects (11.4%) with overt T2DM and we found no association between* H. pylori* serological status and overt T2DM in all racial/ethnic and gender groups in this population (data not shown).

Although the current study showed a much higher prevalence of abnormal glucose tolerance in the seropositive subjects in each racial/ethnic and gender group (Table 2), they were much older. After the consideration of age, BMI, and covariates, the seropositivity of* H. pylori* was no longer a risk factor of abnormal glucose tolerance (Table 3). Our results were consistent with the two reports of no association between* H. pylori* serological status and T2DM from the United States, namely, the Multiethnic Study of Atherosclerosis [9] and the Third National Health and Nutrition Examination Survey [11]. In contrast, the Sacramento Area Latino Study on Aging reported an adjusted hazard ratio of 2.69 for the development of diabetes in the seropositive MA subjects [10]. The main differences in the latter study were as follows: (1) it was a prospective study, (2) a much smaller seronegative sample size (7%, 63 subjects) was identified, and (3) a much older population (60 years or older with a mean age of 69 years old) was enrolled. Contradictory results were also noted from other countries. Most of the positive associations were from the studies with relatively small sample size (<200 subjects) [14–16], except for two studies (250 and 420 subjects) [17, 18], while most of negative association studies were from a much larger sample size (>500 subjects) [19, 20]. Furthermore, based on the histopathological confirmation of* H. pylori *infection, no association was found between* H. pylori *infection and T2DM [21]. Eradication of* H. pylori* infection failed to improve glycemic control in 174 patients with T2DM [4]. Ascertainment of* H. pylori* infection, small sample size, and selection bias could lead to conflicting results. Thus taking this all into consideration, it is unlikely that* H. pylori* infection plays a major role in the pathogenesis of T2DM.

The present study showed no association of* H. pylori* seropositivity with insulin resistance in subjects without diabetes in most racial/ethnic and gender groups (Table 4), except for the NHB female subjects. In contrast, in the NHB female subjects,* H. pylori* seropositivity was associated with less insulin resistance, albeit with only marginal significance (*P* = 0.04) after adjustment for covariates. Association by chance is highly suspected and further confirmation is required. In the three studies from the United States, the serological status of* H. pylori *had no impact on HOMA-IR as noted in the Sacramento Area Latino Study on Aging [10], and no impact on fasting insulin concentration (a segregate of insulin resistance) in the Third National Health and Nutrition Examination Survey [11], while it was not examined in the Multiethnic Study of Atherosclerosis [9]. Thus, no association between* H. pylori* seropositivity and insulin resistance was found in the United States. Although the conflicting results using HOMA-IR were noted from the other parts of the world and most of the studies were relatively small (<100 subjects), five studies demonstrated a higher HOMA-IR in the* H. pylori* seropositive subjects than seronegative subjects [22–26], while two studies showed no difference in HOMA-IR [27, 28]. In a Japanese population,* H. pylori* seropositivity was significantly higher in 99 cases with insulin resistance (HOMA-IR ≥2.5) compared with 1008 cases without insulin resistance (HOMA-IR <2.5) [29]. Eradication of insulin resistance after successful treatment of* H. pylori* infection was reported in one study [3] and improvement of HOMA-IR after eradication of* H. pylori* infection has been noted in another study [25]. However, no improvement in HOMA-IR was reported by another group [30] after eradication of* H. pylori* infection. Thus, it is unlikely that* H. pylori* infection plays a major role in the pathogenesis of insulin resistance.

In contrast to insulin resistance, there is only one study on the association of* H. pylori* serological status with beta cell function. In a study of 288 Chinese men residing in Hong Kong with various states of glucose tolerance, no association was found between* H. pylori* serological status and beta cell function, based on HOMA-B [28]. However, the* H. pylori* serological status was associated with postchallenge blood glucose at 30 minutes in this Chinese population. Based on the latter finding, the authors reported an association of* H. pylori* serological status with beta cell function [28]. In the present study, no difference was found in HOMA-B in all racial/ethnic and gender groups. Thus,* H. pylori* infection has no impact on beta cell function.

The present study provides some unique complimentary features to two published reports from the United States. In both the Multiethnic Study of Atherosclerosis [9] and the Third National Health and Nutrition Examination Survey [11], the negative association of* H. pylori *infection with T2DM was based on the pooled analysis and confirmed by multivariate analysis with the consideration of racial/ethnic groups and gender. As the prevalence of* H. pylori* infection differs among racial/ethnic groups and genders, we analyzed each group separately and our results are in agreement with these two large scale studies [9, 11] that* H. pylori* infection does not play a major role in the pathogenesis of T2DM in the United States. We also confirmed that* H. pylori* infection does not play a major role in insulin resistance in the United States as noted in Third National Health and Nutrition Examination Survey [11]. Furthermore, we provided the first report in the United States of no association of* H. pylori* infection with beta cell dysfunction. In contrast,* H. pylori* infection leads to an increased rate of incident diabetes in a prospective cohort study in the United States [10]. As the latter study only enrolled the elder population (60 years or older) and the prevalence of* H. pylori* infection and T2DM is much higher in the elder population, the role of* H. pylori* in the pathogenesis of T2DM in the elder population requires confirmation in other elder populations. Results of our analysis, however, do not suggest a major role of* H. pylori* infection in the pathogenesis of insulin resistance, beta cell dysfunction, and abnormal glucose tolerance in a large United States cohort.

## Figures and Tables

**Table 1:** Clinical characteristics of studied subjects.

	NHW		NHB		MA	
	Mean	STD (%)	Mean	STD (%)	Mean	STD (%)
*n*	1,949		853		1,334	
Gender (female)	1,000	51.31%	453	53.11%	723	54.20%
Age (year)	50 ± 21		44 ± 19		43 ± 20	
Body mass index (kg/m^2^)	27.56 ± 5.91		29.40 ± 7.64		28.06 ± 5.73	
HbA1c (mmol/mol)	36 ± 10		39 ± 14		38 ± 13	
HbA1c (%)	5.4 ± 0.9		5.7 ± 1.3		5.6 ± 1.2	
*H. pylori* seropositive	451	23.14%	465	54.51%	845	63.34%
Abnormal glucose tolerance	569	29.19%	319	37.40%	412	30.88%

NHW: non-Hispanic whites; NHB: non-Hispanic blacks; MA: Mexican Americans.

**Table 2:** Comparison of clinical characteristics by serological status of *H. pylori*.

*H. pylori *	Gender									
	Male					Female				
	Seronegative		Seropositive		*P*	Seronegative		Seropositive		*P*
	Mean	STD (%)	Mean	STD (%)		Mean	STD (%)	Mean	STD (%)	
Non-Hispanic whites										
*N*	722		227			776		224		
Age (Year)	48 ± 20		61 ± 19		<0.0001	47 ± 20		59 ± 19		<0.0001
Body mass index (kg/m^2^)	27.33 ± 5.28		27.95 ± 5.16		0.12	27.74 ± 6.58		27.27 ± 6.04		0.34
HbA1c (mmol/mol)	36 ± 9		39 ± 12		<0.0001	33 ± 8		37 ± 11		<0.0001
HbA1c (%)	5.4 ± 0.8		5.7 ± 1.1		<0.0001	5.2 ± 0.7		5.5 ± 1.0		<0.0001
Abnormal glucose tolerance	234	32%	100	44%	0.001	162	21%	73	33%	0.0002

Non-Hispanic Blacks										
*N*	171		229			217		236		
Age (year)	38 ± 19		49 ± 18		<0.0001	40 ± 18		49 ± 19		<0.0001
Body mass index (kg/m^2^)	27.56 ± 6.13		27.35 ± 6.22		0.74	30.76 ± 8.24		31.43 ± 8.47		0.40
HbA1c (mmol/mol)	37 ± 10		41 ± 16		0.004	38 ± 14		40 ± 15		0.09
HbA1c (%)	5.5 ± 0.9		5.9 ± 1.5		0.004	5.6 ± 1.3		5.8 ± 1.4		0.09
Abnormal glucose tolerance	54	32%	96	42%	0.03	73	34%	96	41%	0.12

Mexican Americans										
*N*	204		407			285		438		
Age (Year)	38 ± 19		46 ± 19		<0.0001	36 ± 19		45 ± 19		<0.0001
Body mass index (kg/m^2^)	26.64 ± 4.91		27.97 ± 5.03		0.002	28.28 ± 6.27		28.68 ± 6.21		0.40
HbA1c (mmol/mol)	36 ± 10		39 ± 13		0.008	36 ± 12		38 ± 14		0.004
HbA1c (%)	5.4 ± 0.9		5.7 ± 1.2		0.008	5.4 ± 1.1		5.6 ± 1.3		0.004
Abnormal glucose tolerance	60	29%	152	37%	0.05	58	20%	142	32%	0.0004

**Table 3:** Odds ratios for abnormal glucose tolerance based on *H. pylori* status.

			Model 1			Model 2			Model 3		
		*n*	OR	95% CI		OR	95% CI		OR	95% CI	
NHW	Male	949	1.642	1.210	2.227	0.932	0.658	1.318	0.911	0.621	1.336
	Female	1000	1.832	1.319	2.545	1.236	0.845	2.545	1.000	0.642	1.559

NHB	Male	400	1.563	1.031	2.370	0.973	0.582	1.627	0.760	0.416	1.389
	Female	453	1.352	0.922	1.984	0.763	0.473	1.230	0.699	0.393	1.244

MA	Male	611	1.430	0.996	2.054	0.939	0.622	1.416	1.139	0.701	1.848
	Female	723	1.877	1.321	2.666	1.265	0.825	1.940	1.399	0.848	2.309

OR: odds ratio; 95% CI: 95% confidence intervals; NHW: non-Hispanic whites; NHB: non-Hispanic blacks; MA: Mexican Americans.

Model 1: unadjusted; Model 2: adjusted for age and body mass index; Model 3: adjusted for age, body mass index, poverty index, education, alcohol consumption, tobacco use, and physical activity.

**Table 4:** Comparison of HOMA-IR based on *H. pylori* status.

		*H. pylori* seronegative		*H. pylori* seropositive		*P* ^1^	*P* ^2^	*P* ^3^
		*n*	Mean ± SD	*n*	Mean ± SD			
NHW	Male	329	2.871 ± 2.586	90	3.021 ± 1.900	0.607	0.647	0.638
	Female	358	2.563 ± 2.069	93	3.188 ± 4.843	0.061	0.080	0.328

NHB	Male	79	3.119 ± 2.973	99	3.579 ± 3.236	0.329	0.272	0.350
	Female	113	3.616 ± 2.850	88	3.089 ± 2.569	0.176	0.025	0.040

MA	Male	86	2.993 ± 1.663	173	3.279 ± 2.376	0.317	0.751	0.766
	Female	125	3.487 ± 2.939	188	3.361 ± 2.348	0.674	0.899	0.483

SD: standard deviation; NHW: non-Hispanic whites; NHB: non-Hispanic blacks; MA: Mexican Americans.

*P* ^1^: unadjusted; *P* ^2^: adjusted for age and body mass index; *P* ^3^: adjusted for age, body mass index, poverty index, education, alcohol consumption, tobacco use, and physical activity.

**Table 5:** Comparison of HOMA-B based on *H. pylori* status.

		*H. pylori* seronegative		*H. pylori* seropositive		*P* ^1^	*P* ^2^	*P* ^3^
		*n*	Mean ± SD	*n*	Mean ± SD			
NHW	Male	329	129 ± 105	90	118 ± 60	0.382	0.778	0.749
	Female	357	148 ± 130	93	135 ± 132	0.373	0.744	0.889

NHB	Male	79	148 ± 116	99	180 ± 180	0.167	0.092	0.103
	Female	112	216 ± 215	88	323 ± 153	0.467	0.173	0.131

MA	Male	86	153 ± 189	173	141 ± 107	0.526	0.665	0.550
	Female	125	219 ± 192	188	167 ± 102	0.001	0.129	0.733

SD: standard deviation; NHW: non-Hispanic whites; NHB: non-Hispanic blacks; MA: Mexican Americans.

*P* ^1^: unadjusted; *P* ^2^: adjusted for age and body mass index; *P* ^3^: adjusted for age, body mass index, poverty index, education, alcohol consumption, tobacco use, and physical activity.

## Abbreviations

HOMA-B: Beta cell function by Homeostasis Model Assessment
HOMA-IR: Insulin resistance by Homeostasis Model Assessment
MA: Mexican Americans
NHANES: National Health and Nutrition Examination Survey
NHB: Non-Hispanic Blacks
NHW: Non-Hispanic Whites
OR: Odds ratio
T2DM: Type 2 diabetes mellitus.
